# Supplementary material for: Development of an Automated, Non-Enzymatic Nucleic Acid Amplification Test
Source: Micromachines (Basel). 2021 Sep 30;12(10):1204. doi: 10.3390/mi12101204 (PMC8538085; doi:10.3390/mi12101204)
Supplement: Supplementary file 1 [file micromachines-12-01204-s001.zip › micromachines-1374949-supplementary.pdf]

Supplementary Information

Development of an Automated, Non-Enzymatic Nucleic Acid Amplification Test

Zackary A. Zimmers [1], Alexander D. Boyd [1], Hannah E. Stepp [1], Nicholas M. Adams [1] and Frederick R. Haselton [1,2,*]

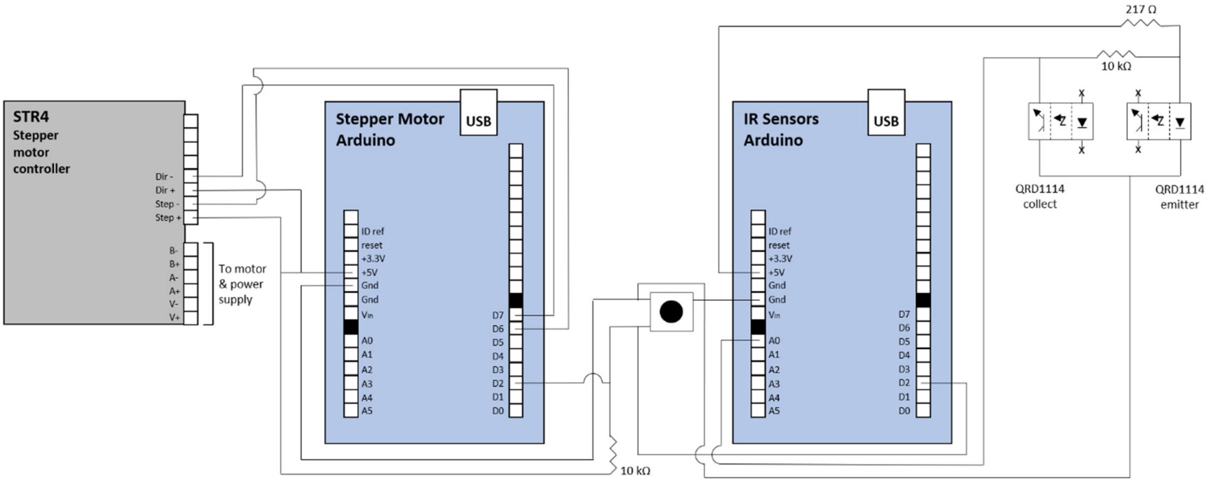

Figure S1. Circuit diagram for the autoPiLOT device. Both Arduinos are connected via USB to the Raspberry Pi B 3+. The button in the center is used at the start of the run to initiate specific portions of the onboard Arduino code.

Effect of buffer and mixing conditions on dumbbell binding.

Several buffer conditions were used to facilitate the binding of dumbbells U1 and U2 to each other, to investigate whether a difference was observed in larger dumbbell-dumbbell product formation. The results, shown in **Figure S2**, show that 2X and 5X SSC buffer make no appreciable difference in dumbbell formation, nor does the addition of Tween 20. To check that the mixing process in the autoPiLOT assay does not disrupt the bonds between dumbbells, U1 and U2 were mixed and incubated for 30 minutes. Magnetic beads were then added and the solution was vortexed vigorously before being loaded into a gel. Lane 6 in **Figure S2** shows that this does not break the bonds between the dumbbells.

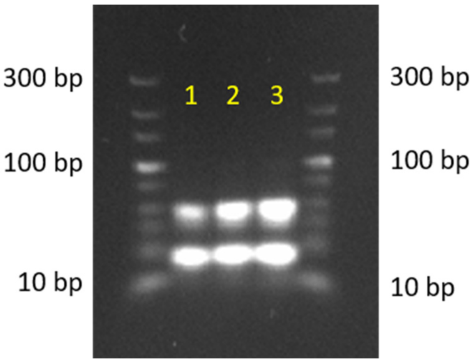

Figure S2. Gel electrophoresis results showing U1+U2 in several buffer conditions. Lanes 1-2: 2X and 5X SSC buffer. Lanes 3-5: 5X SSC with 0.1, 0.05, and 0.025% Tween 20, respectively. Lanes 6 has been vortexed with added magnetic beads just prior to loading the gel.

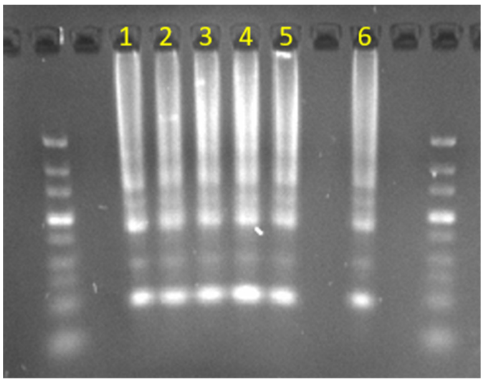

Figure S3. Gel electrophoresis results showing the formation of dumbbell U2 using different ratios of U2-a and U2-b. Lane 1: U2-a:U2-b ratio of 1:2. Lane 2: U2-a:U2-b ratio of 1:1. Lane 3: U2-a:U2-b ratio of 2:1.

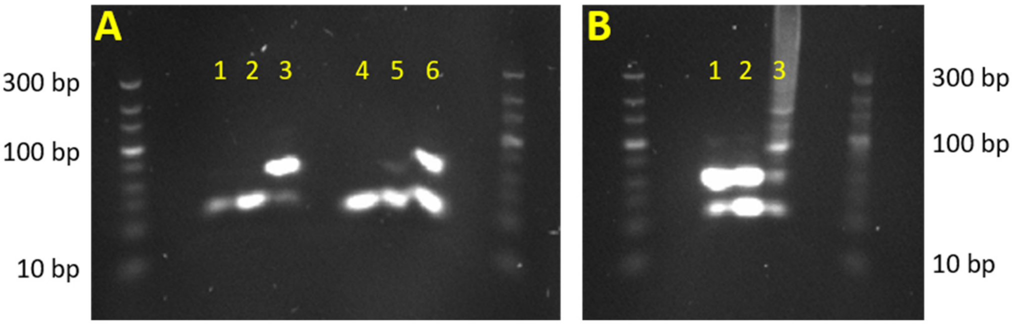

Figure S4. Gel electrophoresis for L-DNA dumbbell binding studies. **A.** Lanes 1-3: U1-a, U1-b, U1. Lanes 4-6: U2-a, U2-b, U2. **B.** Lanes 1-3 show U1, U2, and U1+U2. All L-DNA is labeled with 5′ Texas Red.

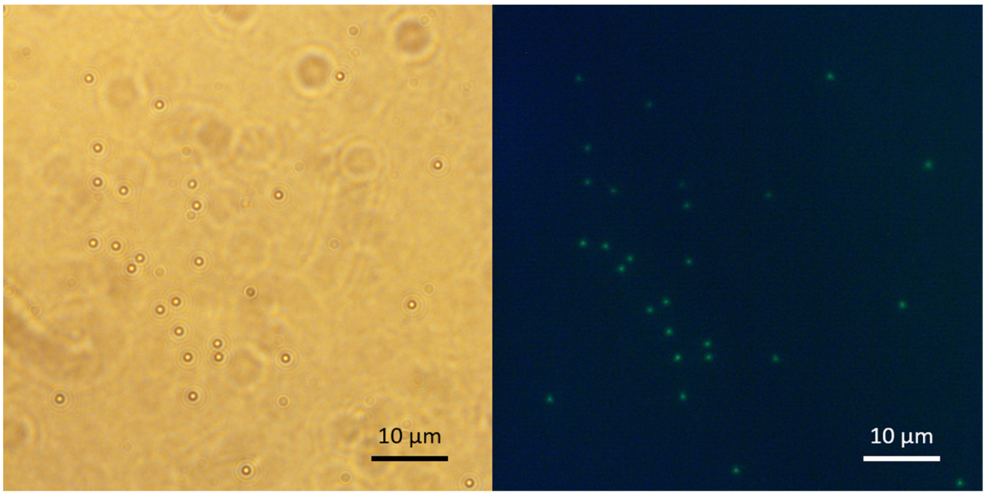

Figure S5. 50X magnification images of magnetic beads which have been incubated in target, followed by FAM-labeled U1*. Left and right images are brightfield and fluorescence images, respectively. The small 1 µM dots are the magnetic beads, visible in both images. The larger, blurry circles in the brightfield image are aberrations caused by dust on the lens.

Effect of magnetic beads on fluorescence measurements.

To achieve real-time fluorescence measurements in the autoPiLOT assay, fluorescence is read directly on the surface of the magnetic beads. A high concentration of magnetic beads may interfere with this measurement, but removing the DNA from the beads would terminate the reaction. To investigate the optical interference of magnetic beads on fluorescence measurements, the fluorescence of a dumbbell U1 at 50 nM was measured with varying amounts of magnetic beads added to solution. The results, shown in **Figure S6A**, show that there is a steep drop in fluorescent signal as the concentration of magnetic beads increases. To determine whether this drop affects the sensitivity of the measurements, fluorescence vs dumbbell concentration curves were created both with and without magnetic beads in solution. The fluorescence curves, shown in **Figure S6B**, have nearly identical slopes, which suggests that while the presence of magnetic beads does decrease fluorescent signal, it does not decrease the resolution with which fluorescent DNA can be detected. 0.50 mg/mL was selected as the magnetic bead concentration for optical measurements in the autoPiLOT.

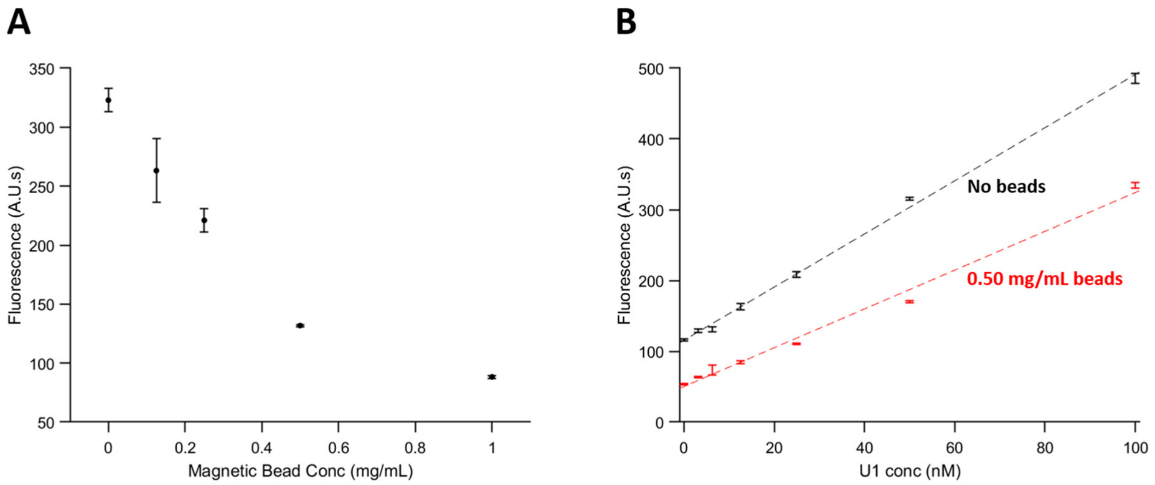

Figure S6. The effect of magnetic beads on fluorescence measurements. **A.** The fluorescence of U1 at 50 nM was measured with varying amounts of magnetic beads added to solution. **B.** Fluorescence curves for U1 ranging from 0-100 nM, both with (red) and without (black) magnetic beads included at 0.5 mg/mL. Error bars depict the mean of three trials ± one standard deviation.

Endpoint removal of DNA from magnetic beads.

There may be applications in which real-time fluorescence measurements are not required, or when the dumbbells bound to the beads must be removed for downstream use. In these situations, a convenient method to remove the dumbbells from the beads would be desired. Perhaps the most straightforward method would be to raise the temperature high enough to melt the DNA, removing all but the biotinylated capture sequence from the beads. This method, however, necessitates equipment to heat the reaction, adding additional components to the autoPiLOT. Alternatively, buffer conditions could be altered such that the DNA melts at room temperature. This can be achieved by raising the pH using NaOH. Finally, a third strategy to remove the dumbbells is via a targeted toehold-mediated strand displacement (TMSD) reaction, as shown in **Figure S7**. This strategy has the advantage of requiring no additional chemical reagents or equipment; a single oligonucleotide can be designed to separate the initial dumbbell U1* from the target DNA. To demonstrate this, a modified version of U1* was created with a 5′ toehold region on U1-a* adjacent to the target-binding domain. This sequence, called U1-a* tag, binds to the sequence U1* removal at the exposed toehold. TMSD then proceeds to displace the target from U1* tag, effectively separating the dumbbell network from the magnetic bead.

All three of the previously mentioned strategies – increased temperature, increased pH, and TMSD – were tested on beads maximally loaded with fluorescently-labeled U1* (or U1* tag for the TMSD test). The beads were incubated in an excess of target, followed by an excess of U1* to load them with fluorescent DNA. They were then washed and subjected to either 95 °C heat, NaOH at pH 12, or the U1* removal oligonucleotide at a concentration of 1 μM. As a control, some beads were also simply resuspended in hybridization buffer. The beads were then magnetically separated and the fluorescence of the supernatant was measured. These results are shown in **Figure S8**. Each method removed approximately equal amounts of DNA from the beads, and no statistical significance was found in the differences between findings. Melting DNA at 95 °C could be called the gold standard method; PCR protocols regularly use this temperature to ensure that all DNA is melted. Therefore, the results of **Figure S8** suggest that DNA separation via high pH and TMSD reaction are both highly effective methods for endpoint detection of DNA dumbbells.

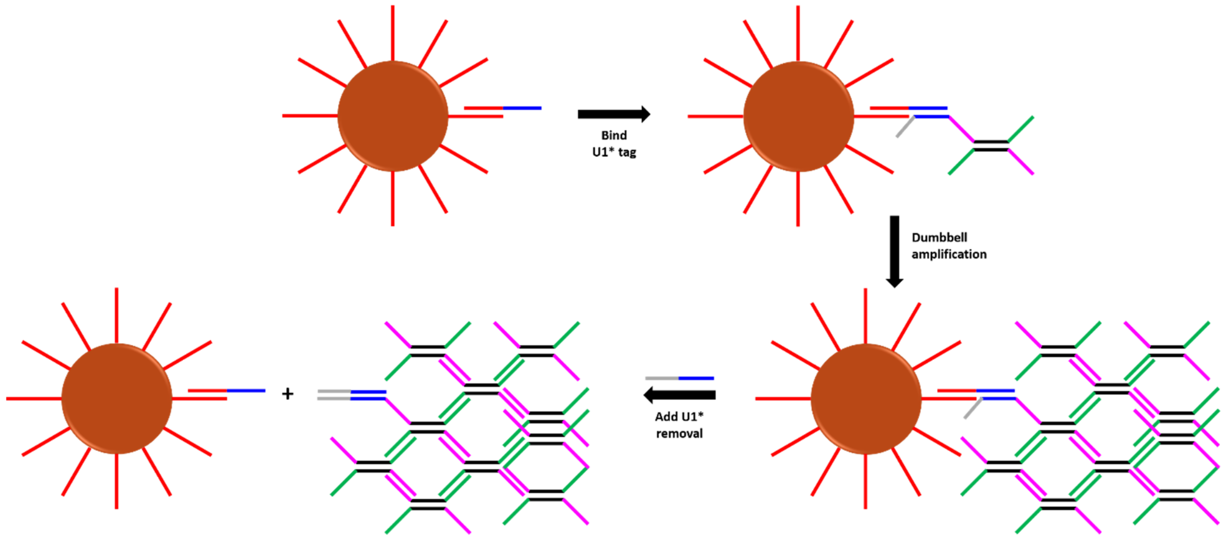

Figure S7. Overview of the TMSD strategy to remove the dumbbells from the magnetic beads. The dumbbell U1* is replaced with a modified version, U1* tag, which has an exposed toehold region (shown in grey). The addition of U1* removal initiates TMSD and displaces the target sequence from U1* tag.

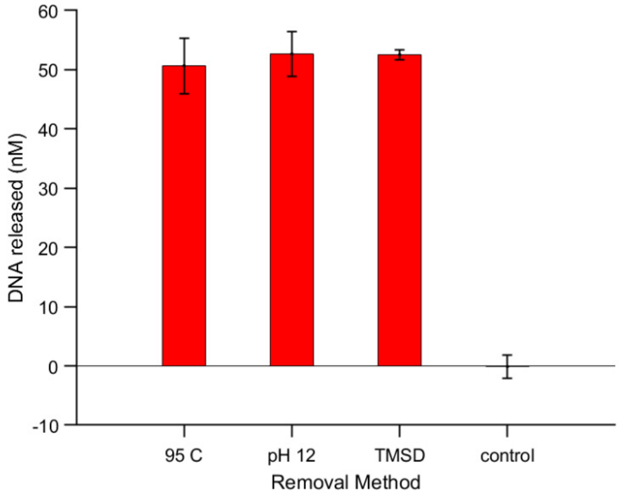

Figure S8. Three different DNA removal methods were tested to separate fluorescent DNA from magnetic beads. Fluorescence measurements were converted to DNA concentration values using fluorescence calibration curves. Error bars depict the mean of three trials ± one standard deviation.

Optimization of bead blocking steps.

There are two means by which non-specific amplification may occur: accumulation on the surface of the magnetic beads, and via fluid carryover between chambers. Even if the non-specific accumulation on the beads is reduced to zero, there will always be some level of fluid transfer between chambers, and therefore some level of non-specific signal amplification[1]. During autoPiLOT amplification, the magnetic beads are cyclically transferred between the two dumbbell chambers many times. Therefore, it is important to minimize both the non-specific binding of DNA by the beads and the non-specific amplification via fluid transfer. To this end, three different bead blocking agents were investigated: bovine serum albumin (BSA), salmon sperm DNA, and biotin. BSA is commonly used as a blocking agent in Western blots and ELISA

assays. Single-stranded salmon sperm DNA is essentially random, with DNA fragments of varying sizes. Biotin binds very strongly to the streptavidin-coated surface of the magnetic beads; it is biotin that anchors the capture probe to the magnetic bead surface. All three of these blocking agents are thought to bind to available surfaces on the beads, decreasing subsequent non-specific binding of DNA dumbbells.

Magnetic beads were functionalized using each of these different blocking agents, as well as without any blocking. Each type of bead was then incubated in fluorescent target DNA. The fluorescence of this solution was measured before and after incubations to determine the fraction of DNA bound by the beads. This was repeated using a different fluorescent DNA sequence, previously published as a malaria PCR primer [2], to measure off-target binding. The results are shown in **Figure S9A**. Biotin blocking produced the lowest average amount of off-target binding, although it was only significantly lower than that of BSA blocking (two-way t-test, $p < 0.1$). Each type of bead was also transported back and forth between dumbbells U1 and U2, to determine whether blocking method impacted the rate of non-specific amplification. The beads were loaded into the autoPiLOT reaction processor, and fluorescence was measured after every other incubation. The results in **Figure S9B** show the accumulation of dumbbells over time on the beads. The results were very similar for unblocked, BSA-blocked, and biotin-blocked beads. Only those blocked with salmon sperm DNA appeared to exhibit significantly higher non-specific amplification. In clinical applications, the blocking protocol likely needs to be tailored to the sample type. For the studies performed here, based on the results of **Figure S9**, biotin blocking was selected as the best protocol.

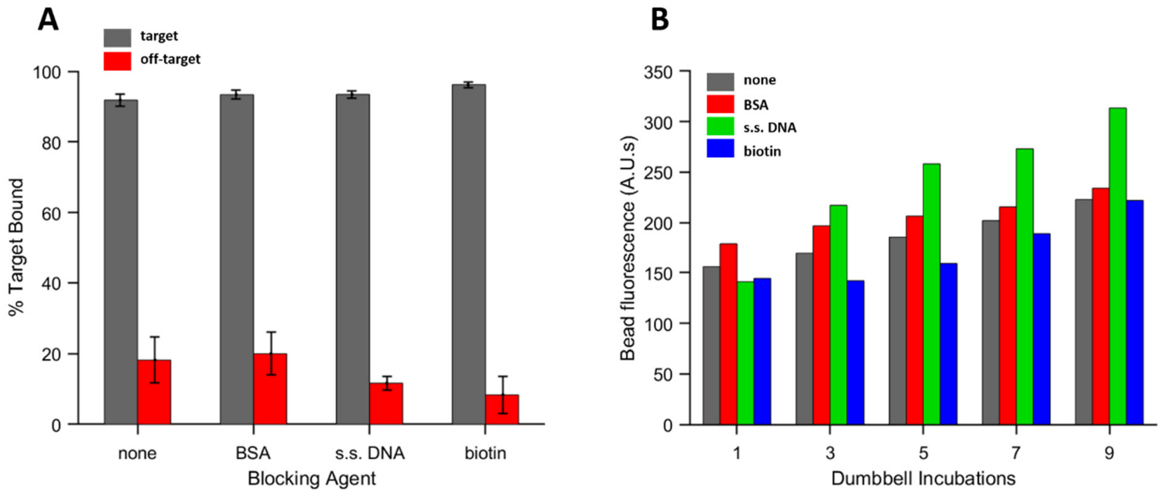

Figure S9. Comparison of different bead blocking protocols on non-specific binding and non-specific amplification. **A.** Target binding (grey) and non-specific DNA binding (red) for beads with different blocking agents. **B.** Each type of bead was used for autoPiLOT assays without any target DNA. The resulting fluorescence represents the non-specific signal amplification.

Effect of dumbbell incubation time on amplification.

The authors who first demonstrated the dumbbell amplification assay used 30-minute incubation times for each dumbbell. Based on this previous report, 30 minutes was used as the incubation time for autoPiLOT experiments. The hands-free nature of the autoPiLOT makes 30-minute incubations no more cumbersome than 1-minute incubations. In situations where time is a more serious constraint, however, there may be a desire for a shorter reaction timeframe. To investigate the effect of shortening the incubation

time, the autoPiLOT assay was performed with incubation times of 30, 10, and 1 minutes per dumbbell. A total of 15 incubations between U1 and U2 were performed, and samples contained 3*10^8 copies of target DNA. Decreasing the incubation time led to decreases in both D-DNA and L-DNA amplification, as shown in **Figure S10**. The ratio of these two signals, or signal-to-noise ratio, decreased with shortening incubation times. Increasing the incubation time, say from 1 to 10 minutes, increases the target-induced amplification more than it does the amount of non-specific L-DNA amplification.

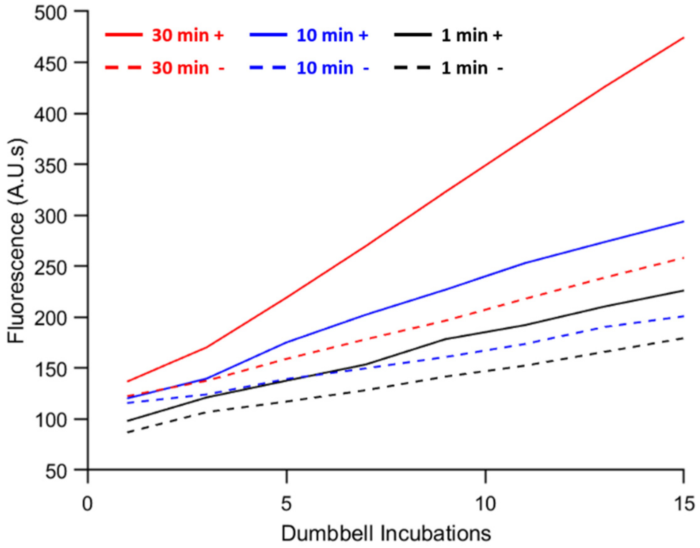

Figure S10. AutoPiLOT amplification trials using 30-minute (red), 10-minute (blue), and 1-minute (black) dumbbell amplification times. Solid lines show D-DNA signal, and dashed lines show L-DNA signal. Displayed values are the mean of three trials.

References

1. Adams, N.M.; Creecy, A.E.; Majors, C.E.; Wariso, B.A.; Short, P.A.; Wright, D.W.; Haselton, F.R. Design criteria for developing low-resource magnetic bead assays using surface tension valves. *Biomicrofluidics* **2013**, *7*, 014104.
2. Bitting, A.L.; Bordelon, H.; Baglia, M.L.; Davis, K.M.; Creecy, A.E.; Short, P.A.; Albert, L.E.; Karhade, A.V.; Wright, D.W.; Haselton, F.R. Automated device for asynchronous extraction of RNA, DNA, or protein biomarkers from surrogate patient samples. *Journal of laboratory automation* **2016**, *21*, 732-742.
